# A dual-tier plasmid network model underpins the evolutionary success of pandemic *Klebsiella pneumoniae* ST11

**DOI:** 10.1038/s41598-026-60651-2

**Published:** 2026-07-22

**Authors:** Alsayed Alfiky, José Manuel Ortiz de la Rosa, Mustafa Sadek

**Affiliations:** 1 Genetics Department, Faculty of Agriculture, Tanta University, Tanta, 31511 Egypt; 2 Institute of Biomedicine of Seville (IBiS), University Hospital Virgen del Rocío/CSIC/University of Seville, Seville, Spain; 3 Department of Food Hygiene and Control, Faculty of Veterinary Medicine, Qena University, Qena, 83523 Egypt; 4 Department of Hygiene and Food Control, Faculty of Veterinary Medicine, King Salman International University, South Sinia, Egypt

**Keywords:** Antimicrobial resistance, Pangenome, Genome evolution, Plasmid epidemiology, Network analysis, Convergent, Carbapenem-resistant hypervirulent *Klebsiella pneumoniae* (CR-HvKP), Global surveillance, Computational biology and bioinformatics, Evolution, Genetics, Microbiology

## Abstract

The convergence of antimicrobial resistance and hypervirulence in high-risk *Klebsiella pneumoniae* clones represents a major public health threat. However, evolutionary mechanisms enabling specific lineages to achieve pandemic dominance remain unclear. In this study, we integrated pangenomics and network analysis across 1,010 complete genomes from 38 countries. Species-wide dynamics revealed an extremely open pangenome (α = 0.59). In contrast, the dominant ST11 lineage, representing 30% of isolates, exhibited extremely low within-lineage phylogenetic diversity, consistent with a recent clonal expansion concentrated in East Asia. The East Asian ST11 lineage exhibited the lowest pangenome diversity (α = 0.86) associated with fixation of persistence and plasmid-stabilization systems and purging of redundant defense mechanisms. This configuration sustains a dual-tier plasmid network comprising a lineage-anchored IncFII(pHN7A8) replicon for vertical stability alongside high-connectivity hubs such as IncFIB(K) facilitating horizontal gene transfer. Chromosomal integration and tandem amplification of key resistance determinants (*blaK*_*PC−2*_, *bla*_*CTX−M−15*_) further reinforced this architecture. Consequently, 34.2% of isolates exhibited convergence of carbapenem resistance and hypervirulence. Within the East Asian ST11 clade, two dominant sub-lineages emerged: KL47:O13 (25.5%) and KL64:O2α (72%). Despite lower IncFII(pHN7A8) penetrance, KL64 became the dominant sub-lineage, indicating that factors beyond plasmid carriage, possibly including surface antigen properties, contribute to its epidemiological success. These findings indicate that ST11 success arises from synergy between species-wide pangenome openness and lineage-specific genomic optimization, and highlight plasmid network topology as a complementary framework for genomic surveillance of adaptive clonal expansion.

**Supplementary Information:**

The online version contains supplementary material available at 10.1038/s41598-026-60651-2.

## Introduction

The spread of multidrug-resistant and hypervirulent pathogens, driven by horizontal transfer of mobile genetic elements (MGEs), represents a critical public health threat. *Klebsiella pneumoniae* stands at the pinnacle of the World Health Organization’s priority list as it transitioned from an opportunistic threat into a global public health priority^1,2^. The convergence of two historically distinct threats, multidrug resistance (MDR) and hypervirulence, into carbapenem-resistant hypervirulent *K. pneumoniae* (CR-HvKP) has created pathogens that defy standard treatment and portend a potential post-antibiotic era^3, 4, 5^. Recent surveillance in Switzerland identified CR-HvKP clones, including ST147-K64, underscoring the ongoing international dissemination of convergent lineages^6^.

While the global spread of specific *K. pneumoniae* clones is documented^7^, the evolutionary mechanisms that enable a single lineage to diversify into multiple successful variants remain unclear. Specifically, the interaction between plasmid ecology and chromosomal evolution in driving intra-clonal diversification and competition remains poorly understood. Furthermore, the mechanisms that enable lineages to stably integrate combinations of lethal traits, while overcoming the fitness costs inherent in complex, multidrug-resistant payloads, have yet to be defined^8, 9, 10^. The global epidemiological landscape reveals a significant evolutionary bottleneck where a stark asymmetry exists between the immense variety of circulating plasmids and resistance genes, and the small number of successful lineages capable of sustaining global transmission, suggesting that only specific genetic backgrounds, such as the notorious ST258, ST11, ST15 or ST23 clades, possess the requisite host-plasmid compatibility to anchor and disseminate these lethal traits^11, 12^. This suggests that successful epidemic clones are not passive recipients of MGEs; rather, they have evolved into optimized genomic ‘hubs’ capable of maintaining specific plasmid repertoires with minimal fitness burden^13^.

Many foundational studies have established the role of broad-host-range plasmids in disseminating resistance genes and noted their persistence in specific lineages^14, 15, 16, 17^. The concept of ‘epidemic resistance plasmids’ (those with enhanced capacity for dissemination among high-risk clones) was first systematically articulated by Carattoli (2009), who classified Inc groups by their host range and epidemic potential. This framework was later applied to *K. pneumoniae*, where IncF plasmids were shown to play a central role in the success of ST258^18^. More recently, an investigation in a regional cohort identified IncFII-like plasmids as primary vehicles for the dissemination of *bla*_*KPC−2*_ within the high-risk ST11 clones^19^. However, a system-level understanding of the principles governing these plasmid-clone associations remains incomplete. The global evolutionary trajectory of this partnership and the network architecture that prevents metabolic collapse during such massive clonal sweeps have yet to be resolved. Unraveling these principles is essential to move surveillance from reactive, gene-centric cataloguing toward a proactive, framework-driven field. 

In this study, we tested the hypothesis that the rise of convergent CR-HvKP is not stochastic but is orchestrated by deterministic interactions between high-fitness chromosomal scaffolds and structured plasmid networks. Our aim was to identify the evolutionary principles and specific genomic signatures that enable high-risk lineages to stably maintain resistance–virulence combinations, thereby flagging priority threats for focused intervention.

## Methods

### Genome acquisition

A total of 1010 complete *K. pneumoniae* genomes (Supplementary Data S1) were retrieved from the NCBI Genome Database (accessed December 2025) using the following selection criteria: (1) Assembly level: We restricted our analysis to circularized, complete genomes to ensure the highest resolution of the plasmidome and to accurately characterize the co-occurrence of virulence and resistance loci, which are often fragmented or misassigned in draft assemblies; (2) Release date: 2023–2024 inclusive; (3) Annotation source: Only genomes processed via the NCBI RefSeq pipeline were included to eliminate annotation bias and ensure comparability across the pangenome; (4) Exclusion of atypical genomes and metagenome-assembled genomes (MAGs).

### Multi-locus sequence typing (MLST)

Genomes were assigned STs using the MLST tool (v2.11) through nucleotide BLAST against the *K. pneumoniae* seven-locus scheme (housekeeping loci: *gapA*,* infB*,* mdh*,* pgi*,* phoE*,* rpoB*,* and tonB*)^20^. For 81 isolates (8%) that remained unassigned due to imperfect allele matches, we employed Kleborate (v3.2.4)^21^, which performs whole-genome MLST by aligning the same seven loci directly to the genome assembly and assigning the closest matching ST, resolving all previously untyped isolates. The complete ST assignments for all 1,010 genomes from both methods are provided in Supplementary Data S1. In addition to resolving typing ambiguities, Kleborate was used to profile the capsular (K) and lipopolysaccharide O-antigen (OCL) loci.

### Pangenome construction and analysis

To define the genomic content of the 1,010 *K. pneumoniae* isolates, we used Panaroo v1.5.2^22^ using raw GFF3 files sourced from NCBI RefSeq. We utilized a strict graph-based clustering approach [clean-mode strict] to mitigate the impact of assembly artifacts. Orthologous groups were defined using a 95% sequence identity threshold, and to maximize phylogenetic resolution, we applied [remove-invalid-genes] and [refind-mode] strict parameters, ensuring the recovery of core genes potentially missed by initial automated annotation. The pangenome was partitioned into core (≥ 99% of isolates), soft core (95–99%), shell (15–95%), and cloud (< 15%) genomes. The pangenome openness was estimated using Heaps’ Law (*n* = *κ N*^*−α*^), where *n* is the expected number of new genes and N is the number of genomes sampled. An exponent α < 1 was considered indicative of an open pangenome trajectory. Calculations were performed using the micropan (v2.1) package in R^23^, and to ensure statistical robustness and eliminate sampling order bias, the α parameter was estimated using 500 random permutations of the isolate sequence order.

To ensure that our results were not driven by overrepresentation of highly related outbreak clones, we performed a phylogenetic dereplication on the EA ST11 clade (*n* = 275). All genomes were compared using Mash v2.3 (sketch size 1,000); pairs with a Mash distance < 0.0001 were considered redundant. A graph-based approach identified connected components, and one representative genome per component was retained. This reduced the EA ST11 dataset to 196 unique representative genomes (71.5% of the original). Heaps’ Law analysis was repeated on this dereplicated subset to verify the robustness of the lineage-specific evolutionary signals.

### Comparative pangenome enrichment analysis on defense systems

Targeted enrichment analysis focusing on defense systems and plasmid stability modules was performed to compare the ST11s of the EA clade (*n* = 275) against a diverse cohort of (transcontinental) ST11 isolates (*n* = 29) to identify potential genomic drivers of specialization. Candidate genes were identified through keyword-based mining of pangenome annotations, targeting toxin-antitoxin systems, restriction-modification modules, CRISPR-Cas systems, and plasmid partitioning proteins. Statistical significance for this analysis was assessed using a two-sided Fisher’s Exact Test, and to account for the presence of potential zero-count in gene distributions (e.g., complete gene loss in the EA ST11 clade), the Haldane-Anscombe correction was applied by adding 0.5 to all cells of the 2 × 2 contingency table for the calculation of Odds Ratios (OR) and 95% Confidence Intervals^24^. Multiple testing correction was performed using the Benjamini-Hochberg (BH) method, with a False Discovery Rate (FDR) threshold of < 0.05 considered significant. Genes were classified as EA-Fixed if they exhibited an EA frequency > 70% and an OR > 4, while EA-Losses were defined by a global frequency > 35% and an EA frequency < 10%.

### Phylogenetic reconstruction

Phylogenetic relationships were inferred from a core-gene alignment consisting of 3760 strictly conserved genes (totaling 4,053,367 bp) generated by Panaroo. Maximum Likelihood (ML) reconstruction was performed using IQ-TREE v3.0.1^25^. To ensure computational efficiency without sacrificing accuracy for the 1010-genomes, we utilized the General Time Reversible model with Gamma rate heterogeneity (GTR + G) and the -fast search option. The resulting tree was midpoint-rooted and visualized using the Interactive Tree Of Life (iTOL) v6 (https://itol.embl.de/), with metadata layers integrated to display Sequence Type (ST), geographical distribution, and Kleborate-derived resistance and virulence scores.”

Phylogenetic diversity (PD) was quantified using Faith’s PD, calculated as the sum of all branch lengths in the phylogeny^26^. To characterize the expansion of the ST11 lineage, the tree was pruned to include only ST11 isolates using the keep.tip function in the ape (v5.8) package in R (v4.3.3), and to account for differences in sample size across lineages, we performed rarefaction analysis using 100 permutations per sampling step via the picante package. The relative contribution of ST11 to the species-wide diversity was determined by the ratio of ST11-specific PD to the total tree PD. To further quantify the saturation of these diversity curves, we calculated the rate of phylogenetic discovery (slope) at the final sampling interval; a saturation coefficient (the ratio of final to initial discovery rates) was used to confirm that the sampling depth was sufficient to capture the lineage’s evolutionary breadth. Finally, to normalize for sample size difference between ST11 (*n* = 304) and ST23 (*n* = 43), we calculated a normalized PD metric (per-isolate *PD*_*norm*_) defined as the total Faith’s PD (sum of all branch lengths) of the lineage-specific subtree divided by the number of isolates in that lineage (*PD/n*)^27^.

### Antimicrobial resistance gene identification

Antimicrobial resistance (AMR) gene identification was conducted using the ResFinder v4.7.0, chosen for its proven accuracy in detecting acquired AMR genes from whole-genome data and its regularly updated database^28, 29^. Analysis was performed with default parameters balancing sensitivity and specificity (minimum template coverage of 60% and a nucleotide identity threshold of 90%) against the locally mirrored, curated database ResFinder_db (updated 2025-09-09) from the Center for Genomic Epidemiology repository, hosted on Bitbucket. All genomes were processed using a batch-processing approach to ensure consistency across the dataset, and for each genome, acquired resistance genes were identified by searching using the following parameters: -ifa to specify the input FASTA file, -s “*Klebsiella*” to define the species-specific search parameters, and -acquired to focus specifically on acquired AMR determinants. This workflow generated individual output directories per genome, containing tabular and JSON files with gene calls, alignment statistics, and overlap resolution notes. ResFinder outputs were then merged into a clean summary table, and to ensure the high fidelity of the identified resistome and avoid the inclusion of fragmented or non-functional sequences, raw results were subjected to strict filtering where matches were only retained if they exhibited a nucleotide identity of ≥ 95% and a minimum template coverage of ≥ 90% relative to the reference gene length. Out of the initially identified matches (18,752), only (16,823) high-confidence determinants passed these criteria and were used for subsequent prevalence and plasmid-linkage analysis.

### Resistance gene filtering

To focus exclusively on plasmid-borne resistance determinants, we leveraged the curated molecule designations ([chromosome] or [plasmid]) within the FASTA headers of the Complete RefSeq assembly records, which are highly reliable for closed, finished genomes^30^. We filtered the dataset (16,823) by contig description: rows where the contig field contained the keyword *“*plasmid*”* were retained, while those labeled “chromosome” were excluded. This yielded a plasmid-only subset of (10,625) rows (63% of total hits). Chromosomal integration events of carbapenemase gene (*bla*_*KPC−2*_) as well as on the extended-spectrum β-lactamase gene *bla*_*CTX−M−15*_ were manually verified by extracting the relevant contigs and analyzing them via MOB-suite v3.1.9^31^. For *bla*_*CTX−M−15*_, the comparative analysis of chromosomal and plasmid hits was performed on two sets: (1) all 94 *bla*_*CTX−M−15*_ chromosomal hits, and (2) a random representative subset of the plasmid-borne contigs (*n* = 30, out of 219) as a control group. This random subset was sufficient to establish the highly significant differences in GC content and contig size (*p* < 0.001; Table 5); analyzing all 219 plasmid contigs would not have altered the conclusions and was therefore unnecessary. Analysis of genomic features, specifically GC content (%) and contig size (Mb), was performed using Welch’s t-test to determine statistical significance. A *p*-value of < 0.05 was considered statistically significant, with *p* < 0.001 denoted as highly significant.

### Plasmid replicon identification

Plasmid replicons were identified by querying all genome assemblies against the PlasmidFinder database (Enterobacteriaceae scheme, v2.1.6), and to optimize detection we employed the KMA (K-mer Alignment) algorithm (v1.6.8). The database was indexed using kma index, and mapping was performed using default parameters (≥ 95% identity and ≥ 60% coverage), with the following parameters: -asm (assembly mode), -1t1 (one-to-one mapping), and -cge (CGE-compatible output).

### Plasmid network construction and structural validation

To visualize the plasmid association landscape, we constructed a bipartite network connecting replicons via their shared Sequence Type (ST), applying a weighted projection to evaluate evolutionary compatibility across the *K. pneumoniae* population. To distinguish between physical fusion (where replicons mapped to the same assembly contig) and independent co-residence (where replicons mapped to distinct contigs), we performed a structural validation of the primary hub replicons. Using the KMA-derived fragment mapping files (.frag.gz), we were able to trace each replicon such as IncFIB(K) and Col440I to their specific assembly contigs. This targeted verification confirmed the physical nature of our identified hubs as independent, co-resident plasmids rather than structural fusions.

### Plasmid prevalence and plasmotype analysis

To characterize the distribution of the *K. pneumoniae* mobilome, we calculated the prevalence of each plasmid replicon across the identified sequence types (STs), and to prevent statistical inflation caused by multi-copy plasmids or assembly artifacts, data were normalized at the genome level. A “presence/absence” matrix was constructed where an isolate was scored as positive for a specific replicon if at least one hit was identified via PlasmidFinder. The prevalence percentage (*P*) for a replicon (*r*) within a specific sequence type (*ST*_*i*_) was calculated using the formula:

$$\:P\:(r,\:STi)=\:\left(\frac{{N}_{isolates\:with\:r\:in\:STi\:}}{{N}_{Total\:isolates\:in\:STi\:}}\right)\:\times\:\:100$$

Hierarchical clustering of the prevalence matrix was performed using the Euclidean distance metric to identify stable plasmotype signatures among high-risk clones.

### Plasmid-ST enrichment analysis

To investigate the host-specificity of plasmid replicons, we performed a statistical enrichment analysis between bacterial sequence types (STs) and plasmid replicons identified via PlasmidFinder. For each ST with at least 10 representative genomes (*n* ≥ 10), a 2 × 2 contingency table was constructed to compare the frequency of each replicon within the ST versus its frequency in the remainder of the collection. Statistical significance was determined using a one-tailed Fisher’s Exact Test (alternative = “greater”). To account for multiple testing across the 225 STs and 54 replicons identified, *P*-values were adjusted using the Benjamini-Hochberg False Discovery Rate (FDR) method. Associations were considered significant at an adjusted *P*-value (*P*_*adj*_) < 0.05, and the strength of the association was quantified using the Odds Ratio (OR).

### Phylogenetic stratification and statistical analysis of ST11 sub-lineages

To investigate the distribution and association of replicon IncFII(pHN7A8) within the *K. pneumoniae* ST11 population (*n* = 304), isolates were stratified into sub-lineages based on their capsular polysaccharide (K) and lipopolysaccharide (O) loci, where two primary sub-lineages were defined for comparative analysis: (1) KL64:O2α and (2) KL47:O13. The association was assessed using Fisher’s exact test and the degree of association was quantified using the OR, with the KL47:O13 sub-lineage serving as the reference group; a *p*-value of < 0.05 was considered statistically significant.

## Results

### The expansive pangenome and skewed global population structure

Analysis of the *K. pneumoniae* genome population (*n* = 1,010) revealed a highly dynamic pan-genome consisting of 31,031 gene clusters and a highly expansive pangenome architecture. Mathematical modeling of the pangenome accumulation using Heaps’ Law confirmed an open pangenome state with an “α” value of 0.5919 (Intercept = 765.09). This value characterizes our *K. pneumoniae* population as having a strongly open pangenome, with an essentially unlimited capacity for gene acquisition.

**Fig. 1:**
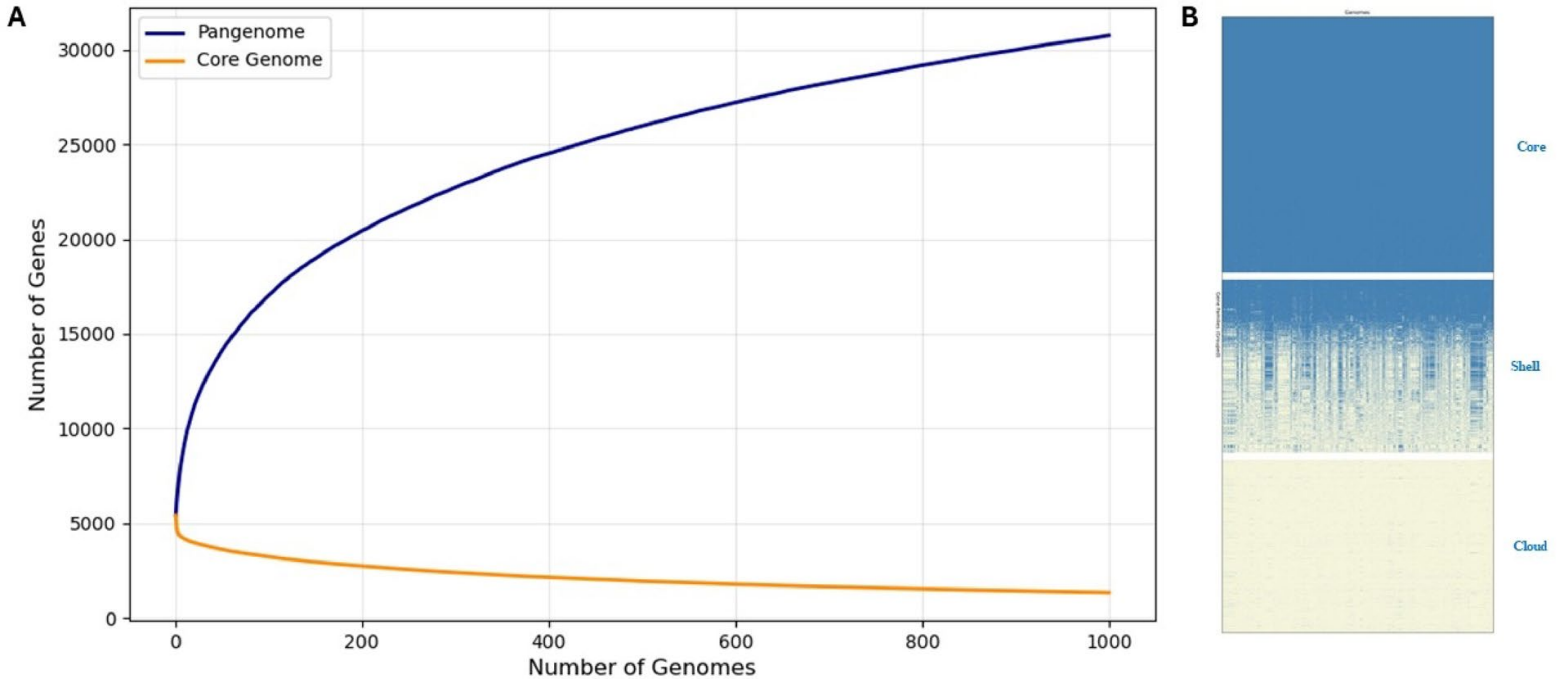
Pan-genome analysis of *K. pneumoniae* (*n* = 1,010). (**A**) The accumulative-curve for the genomes of *K. pneumoniae*. The curve represents the size of pan and core genomes as the number of genomes increased. Pangenome dynamics analysis (Heaps’ Law) confirms an open pangenome architecture (α = 0.5919), highlighting the continuous acquisition of fitness-conferring elements within the accessory genome. (**B**) Presence/absence matrix of the genes identified where the pan-genome is subdivided into core, shell and cloud genomes.

The lack of saturation in gene discovery (Fig. 1) underscores the significant genetic plasticity of the species.”

**Fig. 2:**
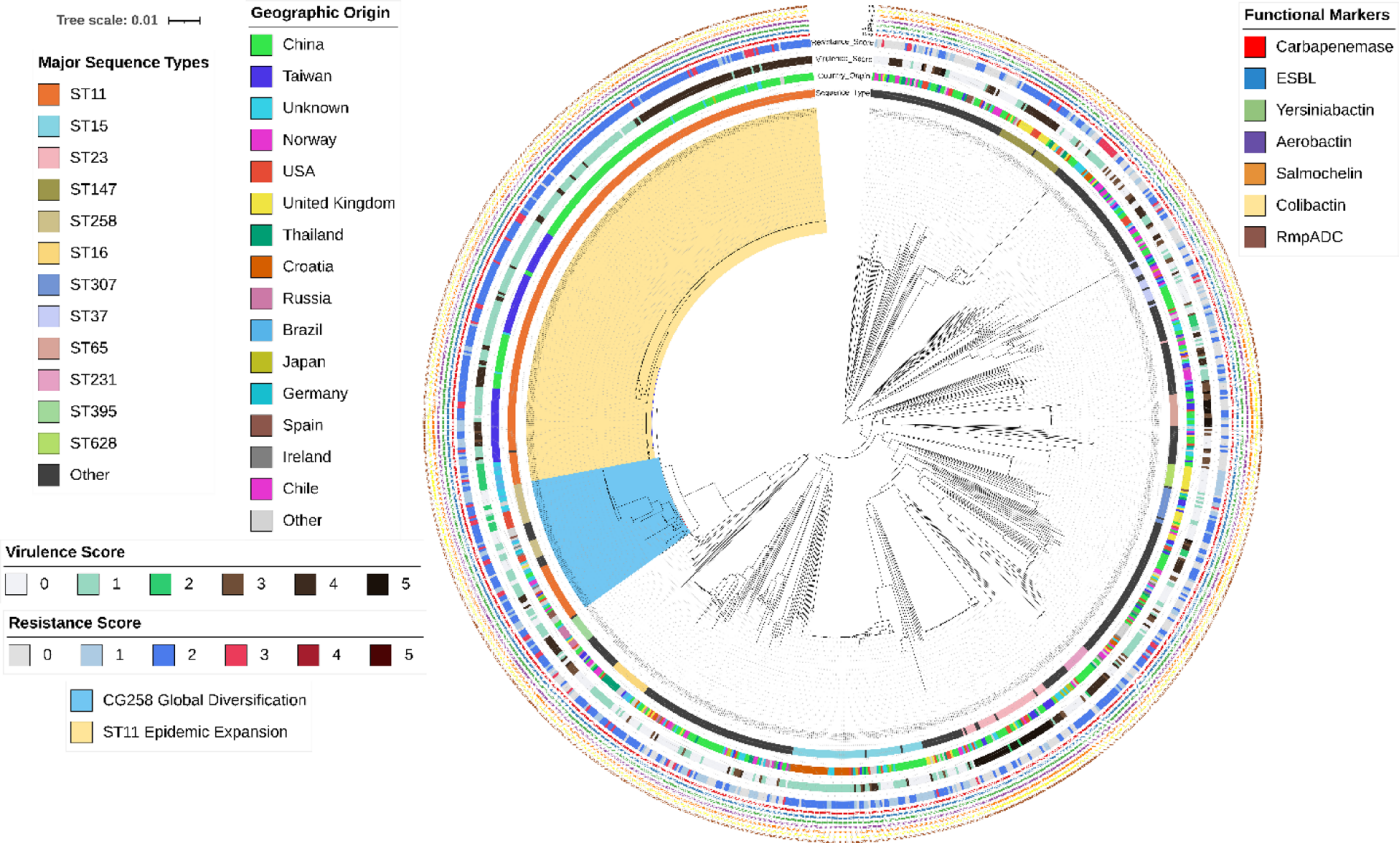
Core-genome phylogeny and phenotypic convergence of *K. pneumoniae* isolates (*n* = 1,010). Maximum-likelihood phylogenetic tree reconstructed from core-genome SNPs identified via Panaroo. The metadata rings, from innermost to outermost, indicate: (i) Sequence Type (ST); (ii) geographic origin; and (iii) standardized resistance and virulence scores (range 0–5). This visualization highlights the convergence of multi-drug resistance (MDR) and hypervirulence (hv) across the population. The aligned heat map displays the presence (filled) or absence (empty) of key functional markers. Note the dense co-occurrence of *bla*_*KPC*_ and *iuc* within the highlighted ST11 lineage, signifying the establishment of a stable, high-risk convergent phenotype.

Analysis of the pangenome across the 1,010 isolates revealed a stable core genome of 3,760 genes (representing 12.1% of the total pangenome). The “soft core” and “shell” genomes comprised 504 and 2,045 genes, respectively. Notably, the “cloud” genome was remarkably expansive, containing 24,722 genes (79.7% of the total pangenome). This genetic potential manifested in a highly skewed population structure as we identified 239 distinct sequence types (STs), yet diversity was dominated by a few successful clones. The top five most prevalent STs (ST11, ST15, ST23, ST147, ST258) accounted for 46.7% (*n* = 472/1010) of all isolates, while the 15 major STs (≥ 10 isolates) together represented 60.3% (*n* = 609/1010). The remaining 401 isolates formed a diverse “Other” category, consisting of 224 sporadic STs (**≤** 9 isolates) reflecting a broad genetic background (Supplementary Table S1). The phylogenetic tree derived from the core gene alignment displayed a clear structure that aligned well with the predicted sequence types, as illustrated in Fig. 2.

Our MLST profiling identified ST11 as the overwhelmingly dominant lineage (*n* = 304/1010) representing 30.1% of the collection, establishing it as the focal point of the contemporary *K. pneumoniae* dataset. While standard MLST failed to classify 81 isolates (8%), Kleborate successfully resolved these “unassigned” cases, providing a more granular view of the population. Following ST11, the most frequent lineages were ST15 (6.2%), ST23 (4.3%), ST147 (3.3%), and ST258 (2.9%).

The 1,010 *K. pneumoniae* isolates analyzed in this study originated from 38 distinct countries and territories across six continents, providing a broad transcontinental view of the species’ contemporary landscape, with a particular focus on the East Asian epidemic expansion. The collection was dominated by isolates from 11 Asian countries and regions (*n* = 575), primarily China (*n* = 376) and Taiwan (*n* = 135). European representation was robust, spanning 15 countries (*n* = 237), including major contributions from Norway (*n* = 67), the United Kingdom (*n* = 55), and Croatia (*n* = 37). The Americas contributed 107 isolates from eight countries, led by the USA (*n* = 62) and Brazil (*n* = 17), while the remaining isolates were distributed across Africa (Egypt, Uganda, Tanzania), and Oceania (Australia). Although 8.7% (*n* = 88) of isolates lacked explicit geographic metadata, phylogenetic mapping provided critical ancestral context; specifically, 13.6% of these isolates (*n* = 12) were nested within the ST11 East Asian expansion (Clade II), suggesting a likely regional origin and further reinforcing the massive scale of this epidemic lineage.

### The emergence and expansion of high-fitness ST11 clade

Core-genome phylogeny of the dominant Clonal Group 258 (CG258) revealed a major evolutionary bifurcation (Fig. 2). From a common ancestor (node where *n* = 348; branch length 0.0032), one branch led to a diverse, globally disseminated assemblage of ST258 and related STs (*n* = 72; light blue shade in Fig. 2). This branch maintained a relatively short internal architecture (branch length 0.00024) and encompassed a globally diverse array of ST258, ST512, ST437, and ST11 isolates. This clade exhibited a broad transcontinental distribution across five continents, including Europe (Spain, Ireland), Africa (Egypt, Tanzania), the Americas (USA, Chile, Brazil), and Asia (Japan, Taiwan, China).

The other branch, exhibiting six-fold greater genetic divergence (branch length 0.0015), gave rise to a monophyletic ST11 epidemic clade (*n* = 276; yellow shade in Fig. 2). This clade, while currently concentrated in East Asia (China, Taiwan), showed evidence of international dissemination with nested export clusters identified in Brazil and Japan. The presence of a single ST11-1LV variant nested within this clade further suggests ongoing micro-evolution and diversification during its rapid regional expansion.

The 1,010-genome collection exhibited substantial phylogenetic breadth across the core genome. While the population was numerically dominated by the ST11 lineage, the presence of 239 distinct sequence types (STs) indicates that the landscape of our *K. pneumoniae* dataset remains evolutionarily diverse, rather than being restricted to a few stagnant clonal lineages. To quantify the evolutionary impact of the ST11 expansion while avoiding the confounding effects of deep-branch recombination on cumulative diversity metrics, we compared the average branch lengths across the tree using Mean Phylogenetic Distance (MPD). This analysis revealed a striking contrast between lineage frequency and evolutionary divergence. The global species MPD was 0.1000 substitutions per site, reflecting a diverse background population. In sharp contrast, the ST11 intra-lineage MPD was only 0.0049, representing a ~ 20-fold reduction in average genetic distance. This quantitatively characterizes ST11 (which accounts for 30% (*n* = 304) of the isolate cohort) as a highly clonal, recently expanded lineage.

**Fig. 3:**
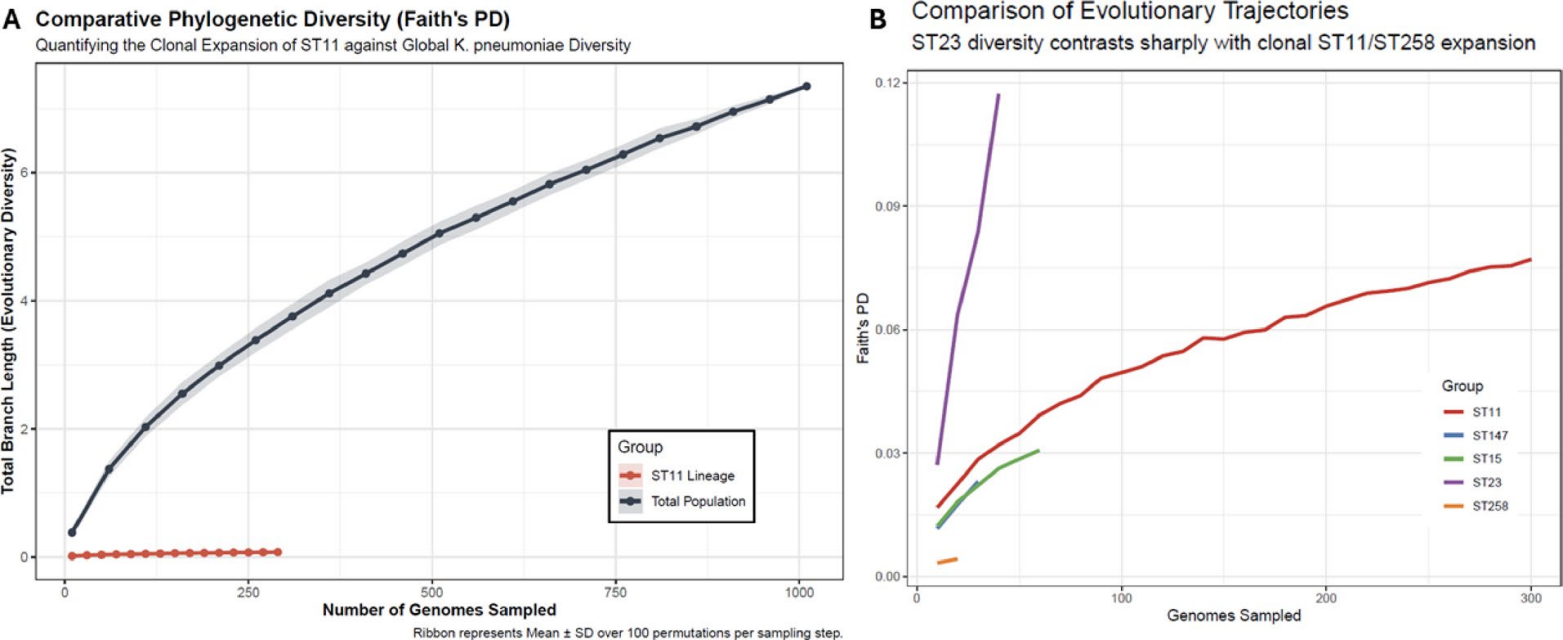
Comparative phylogenetic diversity rarefaction. (**A**) Accumulation curves of Faith’s Phylogenetic Diversity (PD) for the total *K. pneumoniae* population (*n* = 1,010, grey) and the ST11 lineage (*n* = 304, red). Shaded ribbons represent the standard deviation across 100 random permutations per sampling step. The rapid saturation of the ST11 and its significantly lower Mean Phylogenetic Distance (MPD = 0.0049 substitutions/site) compared to the species-wide average (MPD = 0.1000) illustrates a severe phylogenetic bottleneck following the global expansion of this high-risk clone. (**B**) Comparative analysis of lineage-specific evolutionary dynamics highlighting the extreme clonality of MDR lineages (ST11/ST258) relative to the broader diversity of the ST23 lineage, as measured by normalized PD (PD_norm_).

Faith’s Phylogenetic Diversity (PD) analysis provided additional support. Rarefaction analysis of PD confirmed these findings (Fig. 3A); as while the total population curve remained on a steep discovery trajectory, the ST11 curve reached a state of near saturation [(S_c_ST11_) = 0.1381 vs. S_c_Total_ = 0.2064)]. This substantial reduction in phylogenetic breadth, combined with an open pangenome architecture (Heaps’ Law α = 0.592), highlights a population structure where a diverse species-wide reservoir fuels genetic convergence within specialized, low-diversity pandemic clones. Furthermore, we observed a stark divergence among high-risk clones themselves (Fig. 3B), while ST11 was characterized by extreme clonality with normalized phylogenetic diversity (*PD*_*norm*_ ≈ 0.00025), the hypervirulent ST23 lineage exhibited significantly greater phylogenetic depth maintaining over 10-fold higher normalized diversity (*PD*_*norm*_ ≈ 0.0029).

Interestingly, a comparative analysis of pangenome fluidity revealed that while the species-wide pangenome remains highly open (α = 0.592), the ST11 lineage displays the low pangenome diversity expected of a recent clonal expansion (α_total_ = 0.842), reflecting significantly lower genetic diversity compared to the species-wide reservoir. This pattern is most pronounced in the EA ST11 clade, which exhibits both the lowest pangenome diversity (α = 0.866) and a reduced average gene content (Intercept: 814.5) compared to the transcontinental ST11 clade (Intercept: 919.8). Sensitivity analysis using the dereplicated subset of the EA ST11 subset (*n* = 196 unique genomes, **Supplementary Data S1**) confirmed the robustness of this signal, with a Heaps’ Law exponent (α = 0.838) highly consistent with the full cohort (α = 0.866), indicating that the low pangenome diversity is a stable, lineage-specific feature rather than an artifact of sampling bias or localized outbreaks.

Comparative analysis of the EA ST11 clade and the global sporadic ST11 isolates revealed significant differences in the prevalence of defense and plasmid-stabilization systems. Nineteen systems were significantly more prevalent in the EA clade (FDR < 0.05, OR > 4, prevalence > 70%), including for example the PemIK toxin-antitoxin system (OR 35.5) and Hok/Gef post-segregational killing modules (OR 37.2). Conversely, 14 systems were detected at significantly lower prevalence or were absent in the EA clade, including SAM-dependent methyltransferases (65.5% → 0%) and CRISPR-associated Csf2 proteins (Table 1).

**Table 1:** Directional selection of defense systems in East Asian (EA) ST11.

Functional module	System/gene^†^	EA freq (*n* = 275)	Global freq (*n* = 29)	Odds ratio (95% CI)	*P*_adj_ (FDR)
I Positive selection (gains)					
Type II toxin-antitoxin (TA)	*peml/ pemK-mt*	80%	10%	35.5 (9.9–121.5)	< 0.001
Type II TA	HigA	80.4%	17.2%	19.6 (7.2–53.9)	< 0.001
Type I TA	Hok/Gef	81.1%	10.3%	37.2 (10.8–127.5)	< 0.001
Plasmid partitioning	ParA-B	71.6–84.4%	13.8–51.7%	4.5–15.8 (2–46.8)	< 0.01
Antibiotic persistence, toxin	HipA	99.6%	69.0%	123.3 (14.9–1022)	< 0.001
Type II TA	RelE/ParE	94.2%	65.5%	8.5 (3.4–21.3)	< 0.001
Type II TA	VapB/C	77%	34.5%	6.5 (2.8–14.4)	< 0.001
Restriction endonuclease	RE	82.9%	34.5%	9.2 (4.03–21.09)	< 0.001
II Negative selection (losses)					
SAM-dependent methyltransferase	SAM	0%	65.5%	0.001 (0–0.02)	< 0.001
Type II TA Addiction System	PemK/MazF	0%	37.9%	0.003 (0–0.05)	< 0.001
CRISPR-associated protein	Csf2	4.7%	37.9%	0.08 (0.03–0.2)	< 0.001
Type II TA	VapC	0.7%	41.4%	0.01 (0.002–0.05)	< 0.001
Type I TA	Hok/Gef	6.5–6.9%	37.9%	0.12 (0.05–0.29)	< 0.001
Plasmid Partitioning	ParA-B	4.7%	37.9%	0.08 (0.03–0.2)	< 0.001
Type II restriction Endonuclease	RE	4.7%	37.9%	0.08 (0.05–0.2)	< 0.001
Type II TA	RelE/ParE	4.4%	37.9%	0.07 (0.03–0.19)	< 0.001

^†^Where the same functional module appears in both positive and negative selection rows, these represent distinct pangenome gene clusters with the same annotation.

### Sero-resistome convergence and the evolution of a high-fitness KL64:O2α chassis

A striking divergence in surface antigen landscapes was observed between the heterogeneous global ST11 population (*n* = 29) and the specialized EA clade (*n* = 275). While globally circulating ST11 isolates exhibited high serological diversity [8 K-types; primary types KL15 (34%, 10/29) and KL24 (21%, 6/29), while O4 was the most prevalent O-antigen (48%, 14/29)], the EA clade exhibited two dominant sub-lineages. The leading sub-lineage (72%, *n* = 198/275) was defined by a KL64:O2α configuration and a *bla*_*KPC−2*_ penetrance value of 68.7% (136/198). The second sub-lineage carried the KL47:O13 profile (25.5%, *n* = 70/275), and exhibited the highest penetrance of carbapenem resistance, with 91.4% (64/70) carrying *bla*_*KPC−2*_.

Only 11.2% (31/275) of the epidemic clade isolates were carbapenemase-naïve, contrasting with the observed low penetrance (13.8%, 4/29) in the globally circulating ST11 cohort. While *bla*_*KPC−2*_ remained the primary driver of resistance (205/275), the EA clade exhibited remarkable enzymatic plasticity, accommodating 14 distinct carbapenemase variants including *NDM* and *OXA-48*-like alleles. The convergence of triad elements “KL64:O2α:*bla*_*KPC−2*_” observed in 135 isolates is particularly alarming as it combines immune evasion, environmental stability, and antibiotic resistance into a single, highly transmissible package. Carbapenem-susceptible KL64:O2α isolates were also detected in Brazil, indicating international dissemination of this sub-lineage even in the absence of carbapenemase selection pressure.

### Convergence of resistance and hypervirulence on the epidemic scaffold

Within the ST11 lineage of 304 isolates, we observed a striking transition toward a (CR-hvKp) phenotype, defined by the co-occurrence of *bla*_*KPC−2*_ and the aerobactin biosynthesis locus (*iuc* 1 or *iuc* 3) (Table 2). Overall, 30.6% (*n* = 93) of ST11 isolates achieved convergence through the acquisition of the *bla*_*KPC−2*_, and the majority of these (*n* = 88) harbored the *iuc 1* variant. Furthermore, a smaller subset carried alternative carbapenemases including *bla*_*NDM*_, *bla*_*OXA−48*_, and *bla*_*KPC*_ variants (*n* = 11).

**Table 2:** Prevalence of convergent and virulence phenotypes in ST11 (*n* = 304).

Clinical phenotype	Genotype	Count (*n*)	% of ST11	ybt (%)	clb (%)	iuc (%)
Convergent (CR-hvKp)	*bla*_*KPC−2*_ + *iuc 1 / 3*	**93**	30.6	100	0	100
	Other Carbapenemase + *iuc 1*	**11**	3.6	91.0	0	100
MDR, non-hypervirulent	*bla*_*KPC−2*_, *iuc* (-)	117	38.5	98.3	0.8	0
	Other Carbapenemase, *iuc (-)*	42	13.8	90.5	16.7	0
Virulent, susceptible	No Carbapenemase + *iuc 1*	18	5.9	94.5	0	100
Basal ST11	No Carbapenemase, *iuc* (–)	23	7.6	78.3	17.4	0

Furthermore, beyond plasmid-mediated virulence, the ST11 population possessed a chromosomal virulence foundation: 96.1% (*n* = 292/304) of isolates harbored the yersiniabactin (*ybt*) siderophore system, predominantly encoded on Integrative Conjugative Elements ICEKp3 (*ybt* 9). Within the “basal” group in Table 2 (isolates lacking both *iuc* and acquired carbapenemases; *n* = 23), 78.3% (*n* = 18) were armed with *ybt*.

Additionally, we identified a specialized genotoxic sub-population (*n* = 12/304; 3.9%) harboring the colibactin (*clb 3*) locus on the ICEKp10 mega-island. These genotoxic isolates were distributed across the lineage but were notably more prevalent in the basal group (17.4%) and the *bla*_*carb*_ MDR group (16.7%) compared to the *bla*_*KPC−2*_ MDR group (0.85%), highlighting a rare but significant “tripartite” convergence where resistance, hypervirulence (*ybt*), and genotoxicity (*clb*) overlap.

### Universal plasmid penetrance and lineage distribution

Plasmid profiling revealed a vast and deeply integrated mobilome, comprising 2,587 replicons across 58 distinct replicon types. We observed near-universal plasmid carriage, with 95.4% (*n* = 964) of all isolates harboring at least one plasmid replicon (Table S1). This high penetrance extended to the lineage level, where 94.14% (225/239) of identified Sequence Types (STs) were found to be plasmid-bearing, while only 5.86% of STs (*n* = 14) were replicon-free. While the pangenome is saturated with plasmids, the distribution of the mobilome is highly centralized. A small group of four globally recognized high-risk clones (ST11, ST15, ST258, and ST147) collectively sequestered 50.4% (*n* = 1,305/2,587) of the total plasmid replicons, acting as taxonomic sinks for MGEs.

The ST11 lineage demonstrates the capacity for extreme resistance, with individual isolates reaching a peak of 36.0 AMR genes. However, the population exhibits remarkable stability; across 303 replicon-bearing isolates, the mean (16.33) and median (15.0) of AMR gene loads were nearly identical (Table 3, full version Table S2). This population-level stability contrasts with sporadic lineages that occasionally carry much higher plasmid burdens (e.g., a single ST656 isolate with 9 replicons, in contrast with ST11 characterized with (mean 3.1; max 6 replicons), However these hyper-carriers were not epidemiologically successful. We refer to this pattern (the most successful clones are not the most heavily armed) as the intensity-success paradox. These data suggest that while individual isolates across various STs can push the boundaries of genomic capacity, the epidemic success of the ST11 EA clade is underpinned by a highly optimized, population-level genomic architecture rather than maximal gene accumulation.

**Table 3:** Plasmid and anti-microbial resistance burden across major (≥ 10 isolates) and high-risk *K. pneumoniae* lineages.

Sequence type (ST)	No. of replicon-bearing isolates	Total replicons	Replicon types	Range (min-max) replicons	Mean plasmid richness ^1^	AMR Load		
						Average ^2^	Median	Max ^3^
11	303	939	31	1–6	3.10	16.33	15.0	36.0
15	61	151	21	1–5	2.47	19.85	21.0	37.0
147	33	113	19	1–6	3.42	19.88	19.00	31.0
258	28	102	13	1–6	3.64	19.46	21.5	30.0
23	38	65	14	1–7	1.71	5.79	4.00	18.0
307	17	37	12	1–4	2.18	18.76	17.0	30.0
16	17	50	15	1–6	2.94	19.65	20.0	26.0
65	16	30	10	1–3	1.88	6.94	6.0	12.0
37	15	33	17	1–4	2.20	14.93	13.0	30.0
231	14	37	5	1–4	2.64	18.14	18.0	22.0
395	13	40	11	2–5	3.08	16.46	15.0	26.0
628	11	11	1	1	1.00	20.91	21.0	21.0
517	10	33	6	3–4	3.30	5.90	5.0	14.0
392	10	30	4	2–4	3.00	20.90	21.0	22.0

^1^Average number of replicons found in the genomes of that ST (Total replicons/No. of isolates).

^2^Average number of unique Antibiotic Resistance genes found in that ST.

^3^Maximum plasmid count observed in any genome of that ST.

### Lineage-specific ‘plasmotypes’ and the architecture of the plasmid network

**Fig. 4:**
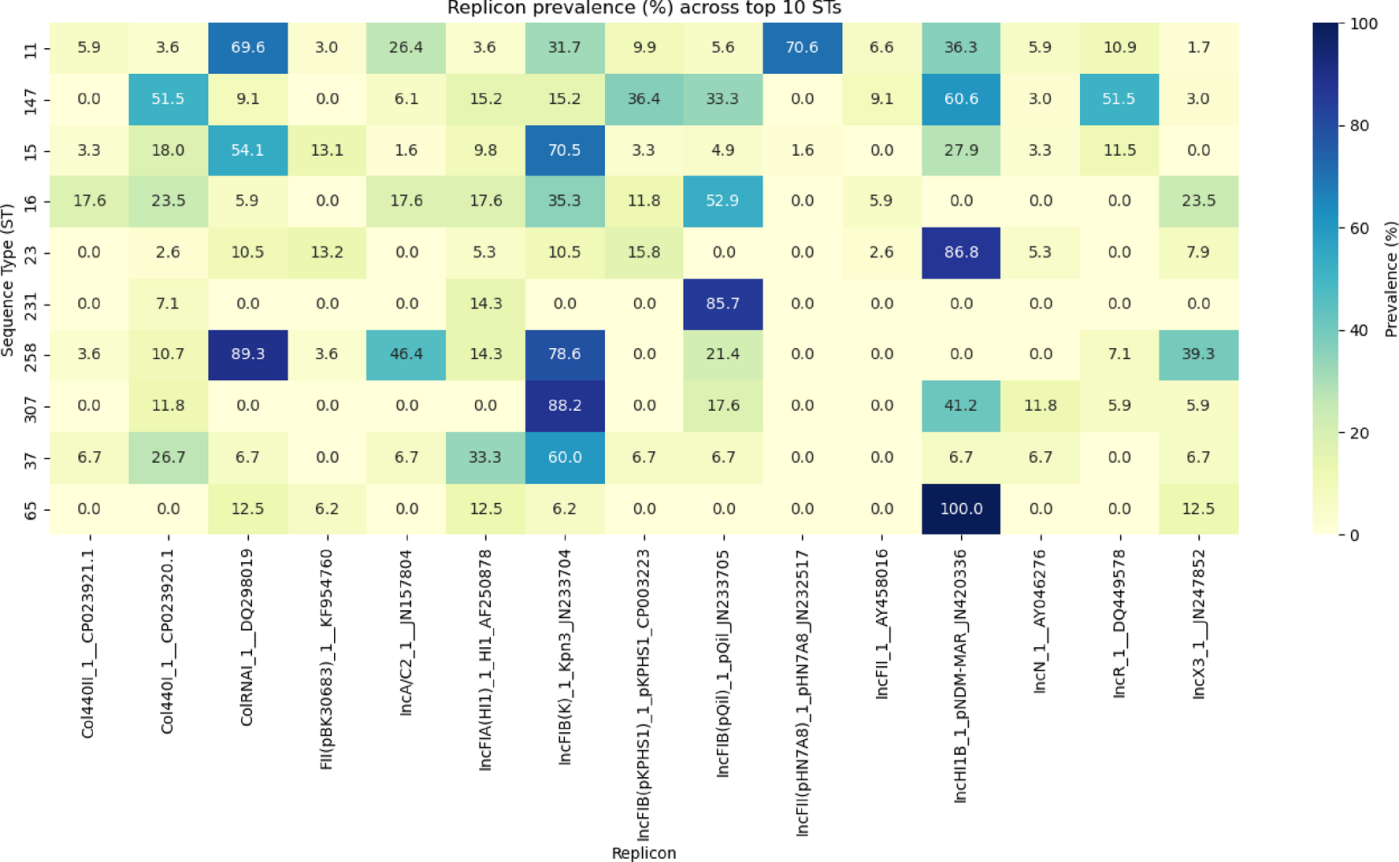
Lineage-specific plasmid ‘plasmotypes’ and replicon tropism. Heatmap illustrates the prevalence (%) of the 15 most frequent plasmid replicons across the 10 dominant *K. pneumoniae* sequence types (STs). Percentages are calculated as the number of isolates within each ST harboring the specific replicon, normalized by the total number of plasmid-bearing isolates in that lineage. The color scale indicates prevalence from 0% (bright yellow) to 100% (dark blue). Hierarchical clustering reveals distinct “plasmotype” signatures, distinguishing stable, lineage-restricted backbones (e.g., IncFII(pHN7A8) in ST11) from broad-host-range promiscuous hubs (e.g., IncHI1B and IncFIB(K)).

Using a prevalence-based analysis to account for varying ST frequencies, we identified highly structured plasmotype signatures that define major *K. pneumoniae* lineages (Fig. 4). The global high-risk clone ST11’s mobilome was defined by a near-fixed association with IncFII(pHN7A8) [70.6% prevalence, OR = 224.6; (214/303 replicon-bearing isolates) compared to non-ST11 isolates], while it was virtually absent in other clones [only in 7 of 706 non-ST11 genomes]. This replicon formed a stable scaffold alongside other prevalent replicons like ColRNAI (69.6% prevalence), IncHI1B_1_pNDM-MAR (36.3%), and IncFIB(K) (31.7%). On the other hand, ST258 was characterized by a distinct signature dominated by ColRNAI (89.3%) and IncFIB(K) (78.6%), followed by IncA/C2 (46.4%) and IncX3 (39.3%) while largely lacking the IncFII(pHN7A8) variant seen in ST11, suggesting clonal specialization of plasmid backbones.

**Fig. 5:**
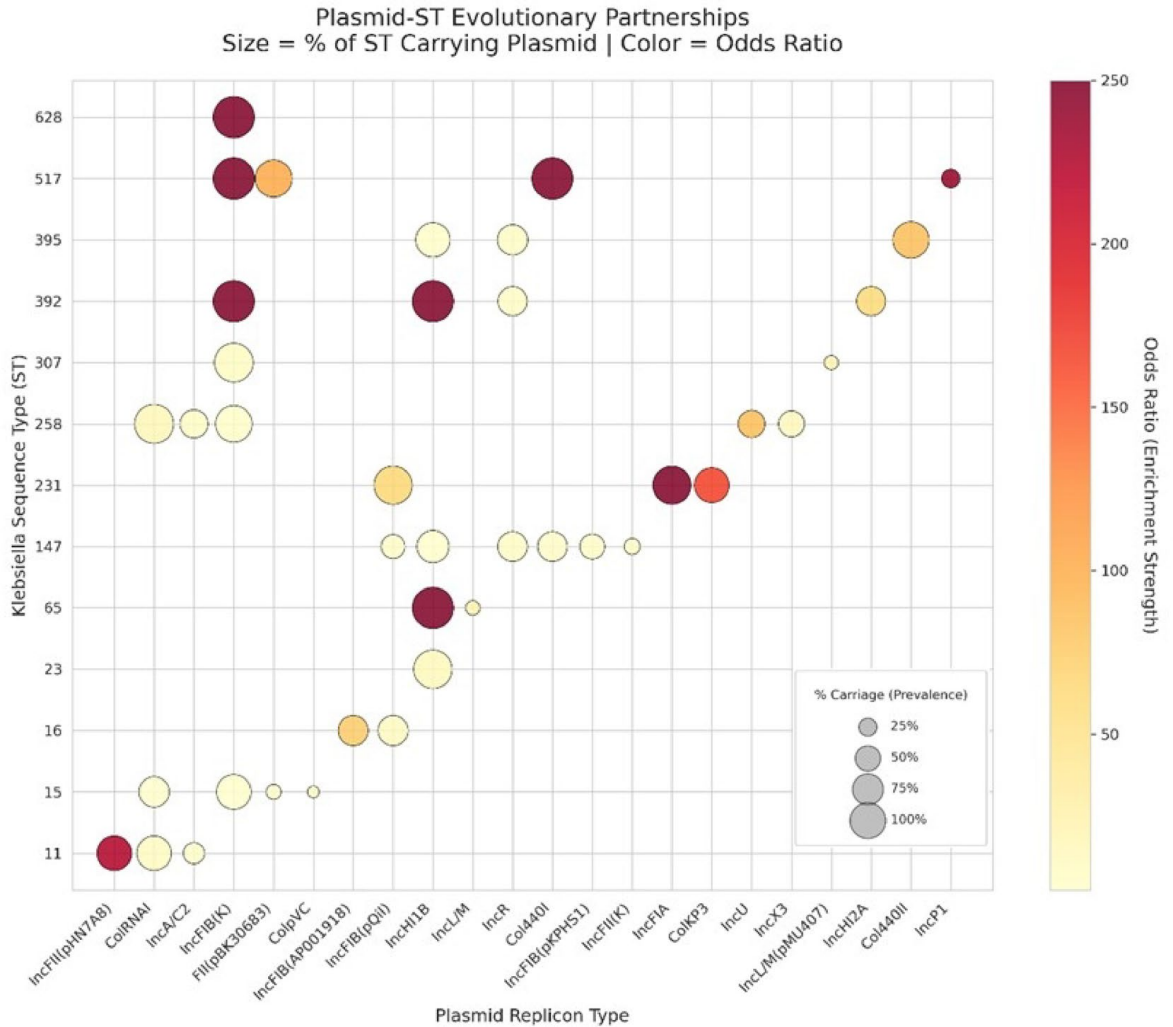
Statistical enrichment and host-plasmid co-adaptation patterns in *K. pneumoniae*. The bubble plot illustrates the strength and significance of associations between major sequence types and plasmid replicons. Bubble size represents the prevalence (percentage of carriage) of the replicon within the specific ST. Bubble color represents the Odds Ratio (OR), indicating the strength of the association (enrichment) compared to the rest of the 1,010 genome collection; values are capped at 250 for visualization. Only associations with an FDR-adjusted p-value (*P*_*adj*_) < 0.05 are shown. The fixation of specific replicons (e.g., Col440I in ST517 and IncFII(pHN7A8) in ST11) highlights stable evolutionary partnerships and lineage-specific plasmid maintenance.

Smaller or emerging lineages showed even higher replicon fixation. Notably, ST231 isolates possessed an 85.7% prevalence of both IncFIA and IncFIB(pQil), and ST16 showed a significant enrichment of IncFIB(pQil) (52.9%; 9/17 isolates). Moreover, IncHI1B_1_pNDM-MAR was particularly dominant in ST65 (100%) and ST23 (86.8%), while ST517 was 100% saturated with Col440I and similarly, IncFIB(K) was 100% prevalent in ST628, ST392 and ST517 (Supplementary Table S3). The IncHI1B (pNDM-MAR) replicon is a standout generalist as it appears in the top rankings for ST11, ST23, ST147, and ST15. This high frequency across diverse lineages suggests frequent horizontal gene transfer events. Formal statistical testing of these replicon–lineage associations is presented in the following section (Fig. 5), where 40 associations reached significance (FDR < 0.05).

### Evidence of host-plasmid co-adaptation

Statistical analysis revealed 40 significant non-random associations between specific *K. pneumoniae* lineages and plasmid replicons (*P*_*adj*_ < 0.05, Fig. 5). We observed a high degree of fixation in several lineages; for instance, the Col440I and IncFIB(K) replicons were present in 100% of ST517 isolates, representing a significant enrichment compared to the rest of the population (*OR* = ∞, *P*_*adj*_ < 0.001).

Similarly, ST11, the most prevalent lineage in our dataset (*n* = 303; plasmid-bearing isolates), demonstrated a powerful partnership with the IncFII(pHN7A8) replicon (OR = 224.6, *P*_*adj*_ = 5.9 × 10^− 128^), with a carriage rate of 70.6%. In contrast, other replicons like IncFIB(K) and IncHI1B_1_pNDM-MAR appeared more promiscuous, distributed across multiple STs with lower Odds Ratios. Notably, the hypervirulent-associated lineage ST231 showed a near-exclusive association with IncFIA_1__AP001918 (*OR* = 1894.0, *P*_*adj*_ = 1.9 × 10^− 21^), further highlighting the lineage-specific nature of plasmid maintenance.

### Differential association of IncFII (pHN7A8) replicon with ST11 capsular lineages

Within the dominant EA ST11 clade (*n* = 275), we observed significant heterogeneity in the prevalence of the IncFII(pHN7A8) backbone between its two major capsular sub-lineages. The IncFII(pHN7A8) replicon was near-ubiquitous in KL47:O13 isolates (91.4%; 64/70), whereas it was present in a lower proportion of the KL64:O2α sub-lineage (71.7%; 142/198). Fisher’s exact test confirmed a significant association between capsular type and replicon carriage (*p* = 4.9 × 10^− 4^; OR = 0.24).

### Co-localization and mobilome analysis of AMR determinants

We identified 16,823 high-confidence AMR determinants across 1010 genomes (≥ 95% identity, ≥ 90% coverage). To ensure high stringency, we focused on physical linkage where genes and replicons co-localized on the same assembly contig. This confirmed linkage in 810 isolates (84.0% of plasmid-bearing strains), while the remaining cases represented chromosomal intrinsic resistance (e.g., *fosA*,* oqxAB*,* bla*_*SHV*_). The *K. pneumoniae* resistome exhibited two principal genomic routes for resistance gene maintenance: chromosomal integration and plasmid-borne carriage (Table S4).

Intrinsic resistance was dominated by the *oqxAB* efflux pumps (found in > 80% of isolates) and *fosA6* (68.3%), both of which were exclusively chromosomal, similar to *bla*_*SHV*_ variants, which were detected in ~ 35% of the cohort with 97.3% chromosomal localization. In contrast, for transferable resistance, sulfonamide resistance (*sul1*, *sul2*) and aminoglycoside-modifying enzymes (*aph(3’’)-Ib* and *aph(6)-Id*) showed the strongest plasmid associations (> 93%). Among β-lactamases, *bla*_*TEM−1B*_ was detected in 49.6% of the isolates with a clear tendency for plasmid-borne association (97.4%), clinically significant extended-spectrum β-lactamases (*bla*_*CTX−M−15*_, *bla*_*CTX−M−65*_, *bla*_*CTX−M−14*_) showed strong plasmid preferences (~ 70–95%), and carbapenemases (*bla*_*KPC−2*_, *bla*_*NDM−1*_) were also less frequent (10–25% prevalence; >95% plasmid association), emphasizing their epidemiological importance. Notably, our analysis revealed significant chromosomal integration events in key resistance genes, which, combined with high redundancy ratios, underscore a transition from transient plasmid carriage to stable genomic maintenance of key resistance traits.

### Chromosomal hyper-stabilization and tandem amplification of resistance genes

Notably, while *bla*_*KPC−2*_ was predominantly plasmid-borne, we identified 14 integration events across 9 isolates where the gene had transitioned to the chromosome. These integrations were localized on large chromosomal contigs (mean size: 5.44 Mb; range: 5.28–5.61 Mb) with a mean GC content of 57.32%, consistent with the *K. pneumoniae* chromosomal features (Table S5). Strikingly, this stabilization was non-randomly distributed, primarily affecting high-risk clones including ST11 (*n* = 4), ST258 (*n* = 2), ST15 (*n* = 2), and ST188 (*n* = 1). Within the ST11 lineage, 75% (3/4) of these chromosomal integrants (including the cases of multi-copy amplification) belonged to the dominant KL64:O2α sub-lineage, while the fourth ST11 isolate (KL25:O2α) shared the same O2α lipopolysaccharide antigen.

A definitive finding was the identification of tandem chromosomal gene amplification in two ST11-KL64 isolates. In the isolate GCF_037157705.1, five identical copies of *bla*_KPC−2_ were arranged in a tandem array within a 19-kb region (positions 811,240 to 831,105), with a consistent inter-genic distance of 4,746 bp; and similarly, isolate GCF_037157715.1 harbored a chromosomal duplication (2 copies).

Similarly, investigation of the 94 putative chromosomal *bla*_*CTX−M−15*_ cases (30%) confirmed their distinct genomic signature. The chromosomal origin of these contigs was further supported by their GC content (mean 57.35%), which was significantly higher than that of the *bla*_*CTX−M−15*_-carrying plasmids (51.47%; Welch’s t-test, *p* < 0.001) and consistent with the *K. pneumoniae* chromosome. The chromosomal contigs were significantly larger (mean 5.39 Mb, range 5.17–5.63 Mb), as expected for genomic scaffolds harboring core genome sequences (Table 5).

Intriguingly, in one exceptional case (GCF_027942195.1), the chromosomal scaffold was predicted to be fully conjugative, harboring a MOBH-family relaxase. While the majority of isolates (*n* = 46/68, 67.6%) harbored a single *bla*_*CTX−M−15*_ chromosomal copy, 32.4% of the lineages exhibited gene amplification, with up to four distinct integration sites identified in a single chromosome (ST323-1LV, GCF_043950395.1) arranged in a tandem array, located between positions 4.82 and 4.83 Mb, and each copy was separated by an identical interval of 4,585 bp. Finally, while 76.7% of the plasmids were predicted to be conjugative, 98.5% of the chromosomal integrations were non-mobilizable, confirming the loss of horizontal transfer machinery upon chromosomal integration.

**Table 4:** Comparative genomic characteristics of *bla*_*CTX−M−15*_ integration cases.

Feature	Chromosomal Hits (*n* = 94 in 68 unique genomes)	Plasmid hits (*n* = 30 hits randomly sampled of 219)	Statistical significance
Contig size (Mb)			
Mean ± SD	5.39 ± 0.08	0.19 ± 0.10	*p* < 0.001***
Range	5.17–5.63	0.009–0.46	(*p* = 2.26 × 10^− 16^)
GC content (%)			
Mean ± SD	57.35 ± 0.13	51.47 ± 1.94	*p* < 0.001***
Range	57.06–57.64	47.01–53.23	(*p* = 5.82 × 10^− 72^)

Significant differences were determined using Welch’s t-test (***p < 0.001).

SD: standard deviation; Mb: megabases.

### The plasmid co-occurrence network and hub replicon architecture

**Fig. 6:**
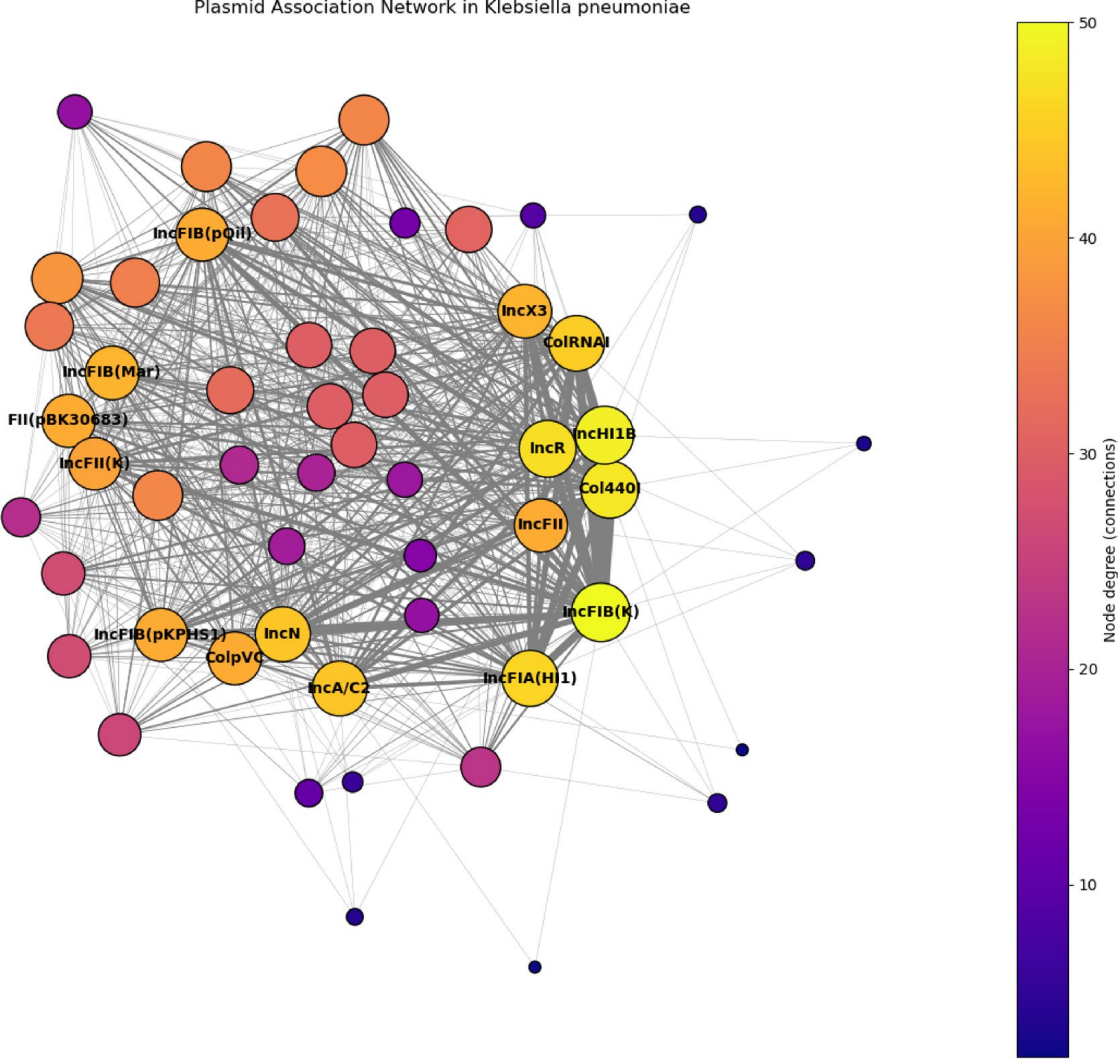
Plasmid association network in *K. pneumoniae.* Nodes represent plasmid replicons connected when they co-occur within the same sequence type (ST). Node size and color scale with degree (number of connections). Labels in the figure indicate plasmid families for clarity, while full replicon identifiers and their exact degree values are provided in Table S6. Centrality analysis identified highly connected replicons such as IncFIB(K), IncHI1B_1_pNDM-MAR, Col440I, IncR, IncFIA(HI1) and ColRNAI acting as hubs (**≥** 45 connections). Edge thickness reflects co-occurrence frequency. Contig-level structural mapping confirms that these co-occurring hub replicons primarily represent distinct, co-resident plasmid molecules rather than structurally fused multi-replicon complexes, illustrating how a small number of compatible hub plasmids likely underpin antimicrobial resistance dissemination.

While chromosomal integration represents a path toward resistance stabilization, the remaining plasmid-borne resistance genes are maintained within a dynamic, highly interconnected network. Analysis of this network for plasmid replicons revealed a complex structure, with 54 plasmids (nodes) linked by 758 co-occurrence edges across STs. On average, each plasmid was connected to ~ 28 others (Fig. 6), indicating that plasmids frequently exhibit high rates of co-occurrence. A small number of replicons, including IncFIB(K), IncHI1B_1_pNDM-MAR, Col440I, IncR, IncFIA(HI1), and ColRNAI acted as hubs, each cooccurring with more than 45 other plasmids, whereas other family members (e.g. IncHI1B(R27), Col(Ye4449) and Col156) remained peripheral (Table S6).

To resolve whether hub-level co-occurrences reflect fusions of multi-replicon plasmids or independent plasmids co-residence, we examined contig-level co-localization of replicon pairs using the alignment fragmentation mapping data. Physical mapping confirmed that these co-occurring hub replicons predominantly reside on distinct assembly contigs. Taking the hub pair IncFIB(K) and Col440I as a representative example, we identified 86 genomes where these hub replicons co-occur. Structural mapping of these instances revealed that 100% of these cases localize to distinct assembly contigs, with zero instances of physical fusion. These results indicate that the strong network connectivity between these two hub replicons primarily reflects the co-residence of distinct plasmids within the same ST, rather than physical linkage on a single plasmid backbone.

## Discussion

The epidemiology of *K. pneumoniae* presents a critical evolutionary paradox: an immensely open pangenome provides near-limitless genetic potential, yet dissemination of multidrug resistance and hypervirulence is driven by the clonal expansion of a few lineages. Our pangenome analysis resolves this paradox by demonstrating that in a species with an extremely open pangenome trajectory [Heaps’ Law α = 0.5919; high ratio of accessory-to-core genes (approx. 7:1)], clonal success is determined not by maximal gene acquisition, but by the lineage-specific optimization of stable plasmid networks. The expansive accessory genome (87.9% of total orthologous groups) confirms the significant genomic plasticity of this collection, providing the substrate for the rapid acquisition of antimicrobial resistance and virulence determinants^32^. Moreover, the observation that four global high-risk clones (ST11, ST15, ST147, and ST258) sequester over 50% of the total plasmid replicon population suggests a non-random distribution of the mobilome, implying that successful lineages serve as stable genomic hubs that have optimized the fitness costs associated with MDR and/or virulence determinants^33^. Ultimately, long-term success of pandemic-potential clones is best explained by a conceptual dual-tier plasmid network model in which specialized plasmid roles provide the necessary balance between alleviating metabolic burden and maintaining an evolutionarily fine-tuned genomic toolkit. This is achieved through specialized plasmid roles: (1) lineage-anchored scaffolds for vertical stability, and (2) promiscuous hubs for horizontal connectivity. Building on the framework of ‘epidemic resistance plasmids’^34^ and its application to *K. pneumoniae*, where IncF plasmids were linked to high-risk clones^18^, our network analysis provides the first population-scale validation of this principle. We quantify the degree of lineage anchoring (OR > 200 for IncFII(pHN7A8) in ST11) and hub connectivity (degree ≥ 45 *for* IncFIB(K) and IncHI1B) that underpins its clonal success. Thus, the broader resistome remains highly fluid, driven by a dense network of plasmid replicons, where key hub backbones like IncFIB(K) and IncHI1B are strongly associated with the dissemination of multi-drug resistance across diverse STs as independent, highly compatible inheritance units rather than structurally fused molecules. However, we note that the co-occurrence network as visualized is dense, and the proposed functional distinction between anchored and promiscuous replicons is inferred from lineage-specific enrichment statistics and contig-level co-localization analysis, not from visually separable network layers.

The explosive rise of the ST11 lineage exemplifies this principle, wherein a clonal high-fitness chassis has stabilized a specialized plasmotype, efficiently orchestrating the convergence of carbapenem resistance and hypervirulence. ST11 dominates our contemporary collection, representing a profound phylogenetic bottleneck; while accounting for 30% of isolates, its intra-lineage mean phylogenetic distance (MPD = 0.0049) is only ~ % of the species-wide MPD (0.1000), quantitatively defining a recent, massive clonal sweep^35^. The success of ST11 is not driven by a higher burden of AMR genes compared to other high-risk clones like ST15 or ST147 (Table 3). Instead, our findings indicate that its widespread dissemination is linked to the maintenance of a stable, lineage-optimized plasmid network we describe herein.

Within this homogeneous backbone, we observe a critical adaptive radiation into two dominant sub-lineages: KL47:O13 and KL64:O2α, providing high-resolution genomic confirmation of the recombination-driven capsule switch previously noted in EA populations^36, 37^. Our data revealed that these are not merely serological variants, but distinct plasmotypes with divergent plasmid profiles. The KL47:O13 variant shows near-saturation of the hallmark IncFII(pHN7A8) replicon (91.4% penetrance) and *bla*_*KPC−2*_ (91.4%), representing a stabilized configuration for carbapenemase maintenance^38^. On the other hand, the KL64:O2α sub-lineage has achieved numerical dominance (72% of the epidemic clade) despite a moderately lower prevalence of the IncFII replicon (71.7% penetrance; OR = 0.24). While this could reflect a shift toward a more optimized fitness chassis in which KL64 relies less on this backbone, an alternative explanation is that the two sub-lineages expanded from ancestors that already differed in their plasmid content, and the observed difference simply reflects their distinct clonal histories. Our genomic data cannot distinguish between these possibilities, and functional experiments would be required to establish whether capsule type directly influences IncFII(pHN7A8) carriage. Nevertheless, the observation that chromosomal *blaKPC-2* integration, including tandem amplification, is specifically enriched in the KL64:O2α sub-lineage provides a potential mechanistic route by which this sub-lineage could reduce its dependence on plasmid-borne resistance, a hypothesis that warrants further investigation. Importantly, this does not imply a reduction in genomic plasticity; our network analysis demonstrates that these traits remain embedded in a dense resistome library, where highly connected hub replicons such as IncFIB(K) and IncHI1B ensure continued access to a diverse gene pool including carbapenemases^39^.

Our proposed dual-tier plasmid network model aligns well with our identification of the IncFII(pHN7A8) replicon as a lineage-anchored scaffold for ST11 (OR > 200) providing population-scale genomic support for earlier mechanistic observations of IncFII-mediated *bla*_*KPC−2*_ transfer^19^. This scaffold minimizes fitness costs and ensures the vertical stability required to underpin the success of the dominant EA-ST11, KL64:O2α sub-lineage^40, 41^. While earlier work used PFGE and PCR to identify these plasmids as ‘good carriers’ in local Chinese cohorts, our application of network theory demonstrates that this association has matured into an epidemically fixed plasmotype. This structural stability is complemented by a second class of promiscuous hub replicons such as IncHI1B_1_pNDM-MAR and IncFIB(K) (Degree ≥ 45), which facilitate horizontal access to the broader mobilome^42, 43^. Our findings provide plausible evidence that resolves the broader "Intensity-Success Paradox". While “genetic sponge” lineages face potential metabolic collapse from extreme resistance and metabolic burdens, pandemic-potential or successful strains thrive by maintaining an optimized, metabolically sustainable burden (e.g., an average of 16.3 AMR genes in ST11)^44^. This suggests that EA-ST11 evolution represents a move toward a modular genomic strategy characterized by the integration of a stable, low-burden anchor scaffold with a high-connectivity horizontal network, to avoid the fitness ceiling of uncurated hyper-carriage while maintaining the evolutionary agility to navigate global clinical environments.

ST11 evolution appears to be shaped by strategic turnover of defense repertoire evidenced by near-fixation of *PemIK* and *Hok/Gef* in the EA clade (OR > 35) and pruning of potentially redundant or high-cost generalist barriers (e.g., SAM-dependent methyltransferases, certain CRISPR proteins). This provides population-scale evidence that ST11 has actively stabilized its plasmid repertoire through addiction systems and genomic optimization^45, 46^. The presence of plasmid stability engines (e.g., Par Systems, TA systems) and observed specific host-plasmid pairing potentially explain the lineage-anchored plasmotypes across STs. Different lineages appear to have evolved a genetic environment in which plasmids are not merely present, but are integrated into the host’s survival strategy through fixed post-segregational killing and partitioning systems^47^. This is complemented by the fixation of HipA, a known driver of antibiotic persistence, which may explain the lineage’s resilience under antibiotic pressure^48^. This framework moves beyond reactive cataloging, offering a mechanistic lens through which we can monitor the metabolic constraints and pangenome ‘openness’ that govern the evolutionary success of emerging high-risk clones.

Upon this optimized scaffold, ST11 is driving an alarming convergence of resistance and virulence which has become fixed in the population and exceptionally difficult to eradicate^49^. While ST11 has historically been the primary vehicle for *bla*_*KPC−2*_ in East Asia^50^, our dataset documents a 34.2% convergence rate (CR-hvKP) within ST11, predominantly through the co-localization of *bla*_*KPC−2*_ and the aerobactin virulence locus (*iuc*). This modular acquisition follows a stepwise evolutionary trajectory: a near-ubiquitous chromosomal virulence foundation (yersiniabactin/(*ybt*), 96.1%), followed by the acquisition of carbapenemase plasmids, and culminating in the plasmid-mediated hypervirulence layer (*iuc*) which illustrates how high-risk clones layer adaptive traits^51^. Our proposed structured network architecture facilitates the efficient convergence of MDR and hypervirulence, creating a pandemic sub-lineage. The presence of isolates carrying alternative carbapenemases (e.g., *bla*_*NDM−5*_, *bla*_*OXA−48*_) with *iuc* (CR-HvKP, ST11), and without *iuc* (CRKP, ST11), represent a reservoir for future convergent clones^37^. The near-universal carriage of yersiniabactin (96.1%), including in the basal group that lacks both *iuc* and acquired carbapenemases, suggests that chromosomal *ybt* is a lineage-defining trait of ST11 that was likely established prior to the widespread acquisition of carbapenemase and hypervirulence plasmids. This finding is consistent with a previous report that all KPC-producing ST11 lineages carry ybt, with a median of 11 ybt genes^3^. The identification of multiple aerobactin variants and diverse ICEKp scaffolds, alongside key resistance chromosomal integrants, signals an advanced stage of resistance stabilization potentially reducing fitness costs and locking resistance into the lineage, which further underscores the high evolvability of the high-risk ST11 chassis^52, 53^. Finally, the presence of the genotoxic colibactin (*clb 3*) in a subset of ST11 clones (3.9%) adds a severe dimension given its ability to induce double-strand DNA breaks in host cells^54^. The identification of a “tripartite” isolate carrying bla_KPC−2_, *ybt*, and *clb* represents a rare but potent convergence of resistance, siderophore-mediated iron acquisition, and genotoxicity, a recipe for a dangerous super pathogen.

Collectively, our findings argue for a paradigm shift in genomic surveillance, moving beyond a reactive cataloguing of resistance genes to proactively tracking high-risk plasmid architectures and their association with successful and evolutionarily optimized clonal chassis. The near-fixed IncFII(pHN7A8)-ST11 association and the observed capsular switch where the dominant KL64:O2α sub-lineage exhibits slightly lower plasmid penetrance are direct outcomes of focused genomic surveillance.

### Limitations

This study has some limitations. First, the geographic distribution of the dataset reflects regional sequencing efforts rather than true global prevalence. The overrepresentation of East Asian genomes means the conclusions regarding ST11 are most directly applicable to that context; dynamics in underrepresented regions (e.g., Africa, parts of South America) may differ, highlighting the need for additional high-quality genomes from these areas^55^. Second, our network analysis is purely genomic; we infer hypervirulence and resistance from genetic markers without phenotype validation through antimicrobial susceptibility testing or virulence assays. Finally, focusing on complete genomes from public databases may oversample prominent epidemic isolates, potentially missing the wider reservoir of diversity that serves as the evolutionary source for emerging clones^56^. Nevertheless, our use of all available 1,010 high-quality contemporary genomes provides the most comprehensive resolution to date of the plasmid ecology underlying the ST11 clonal sweep.

### Future directions

Future work should test whether the KL64 capsular type confers fitness advantages (e.g., in transmission, immune evasion, or environmental persistence) that outweigh the observed difference in plasmid saturation. Longitudinal genomic surveillance, combined with controlled competition assays in vitro and in relevant infection models, will be essential to determine if KL64 is actively displacing KL47 or if they represent stable, niche-adapted strategies. Finally, the accumulating evidence linking ST11-KL64 to increased patient mortality^4, 57^ underscores the urgent need for studies associating capsular types with clinical outcomes. Future prospective investigations should examine whether the distinct genomic architecture of KL64 (particularly its stabilized plasmid network and chromosomal integration of resistance genes) correlates with disease severity, treatment failure, and mortality. Such studies would translate our evolutionary findings into actionable clinical risk stratification.

## Conclusion

The high-risk, pandemic potential in *K. pneumoniae* arises not from a single adaptation but from the evolvability of its lineages and their capacity to maintain a stable genetic backbone while exploring adaptive variants through recombination and plasmid network optimization. This evolutionary flexibility, deciphered through network analysis of contemporary genomes, positions plasmid network architecture as a critical variable for risk stratification in genomic surveillance, providing a framework for interrogating the emergence of future high-risk clones. Our work moves the field toward more precise, hypothesis-driven surveillance and informs the development of targeted interventions against the most stable and threatening pathogen-plasmid combinations.

## Supplementary Information

Below is the link to the electronic supplementary material.

Supplementary Material 1

## Data Availability

The genomic accessions analyzed in this study (*n* =1,010) were retrieved from the NCBI repository; a complete list of accessions and associated metadata is provided in Supplementary Table S1 . All datasets generated and analyzed, including the replicon-ST prevalence matrix, plasmid richness data, and chromosomal integration validations, are included in this published article and its Supplementary Information files. Custom R scripts and analytical pipelines are available via GitHub: (https:/github.com/alsayedalfiky/Kpneumoniae-ST11-Plasmid-Network). Intermediate processed metadata files are available from the corresponding author upon reasonable request.
